# Changes in the microstructure of compact and trabecular bone tissues of mice subchronically exposed to alcohol

**DOI:** 10.1186/s40709-018-0079-1

**Published:** 2018-05-24

**Authors:** Monika Martiniakova, Anna Sarocka, Ramona Babosova, Birgit Grosskopf, Edyta Kapusta, Zofia Goc, Grzegorz Formicki, Radoslav Omelka

**Affiliations:** 1 Department of Zoology and Anthropology, Constantine the Philosopher University in Nitra, 949 74 Nitra, Slovakia; 2 Institute of Zoology and Anthropology, Georg-August University, 37 073 Göttingen, Germany; 3 Department of Animal Physiology and Toxicology, Pedagogical University of Cracow, 30 084 Cracow, Poland; 4 Department of Botany and Genetics, Constantine the Philosopher University in Nitra, 949 74 Nitra, Slovakia

**Keywords:** Alcohol, Microstructure, Compact bone, Trabecular bone, Mice

## Abstract

**Background:**

Alcohol is one of the most commonly consumed neurotoxins by humans. Its negative effect on bone health is known for a long time. However, its impact on qualitative and quantitative 2D characteristics of the compact bone is still unclear. Therefore, the aim of this study was to investigate in detail the effects of subchronic alcohol exposure on compact and trabecular bone tissues microstructure of laboratory mice using 2D and 3D imaging methods. Ten clinically healthy 12 weeks-old mice (males) were randomly divided into two groups. Animals from experimental group (group E; n = 5) drank a solution composed of 15% ethanol and water (1.7 g 100% ethanol kg^−1^ b.w. per day) for 8 weeks, while those from control group (group C; n = 5) drank only water.

**Results:**

Subchronic exposure to alcohol leads to several changes in qualitative 2D characteristics of the compact bone such as the presence of primary vascular radial bone tissue in *pars anterior* of endosteal border and a higher number of resorption lacunae (five times more) in the middle part of *substantia compacta*. Morphometrical 2D evaluations of the compact bone showed significantly increased sizes of primary osteons’ vascular canals (*p *< 0.05) in mice from the experimental group (E group). Sizes of Haversian canals and secondary osteons were not affected by alcohol consumption. In mice from the E group, significantly lower values for relative bone volume and bone mineral density of the compact bone were observed. In the trabecular bone, decreased values for bone volume, trabecular number, trabecular thickness and bone surface (*p *< 0.05) were documented.

**Conclusions:**

Alcohol decreased not only bone volume and density of the compact bone, but it also reduced trabecular bone volume and leads to trabecular thinning. It caused vasodilation of primary osteons’ vascular canals and increased porosity in the compact bone.

## Background

Bone is highly hierarchical in structure, and therefore, any study of its structure and properties must investigate the tissue at several levels of organization in order to gain a complete understanding of the influence of structure and composition on these properties [1]. The extracellular matrix, and especially connective tissue with its collagen (represents more than 90% of the organic bone matrix) confers resistance to the structure and establishes the biomechanical properties of the tissue [2]. Bone mineral (mostly hydroxyapatite) provides mechanical rigidity and load-bearing strength to bone [3]. The relation between organic and non-organic part is crucial for the bone quality [1, 2].

Alcohol consumption is well known to be a lifestyle factor that markedly increases the risk of osteoporosis [4], osteomalacia, aseptic necrosis (primarily necrosis of the femoral head) [5] and fracture incidence [6]. These pathologies are based on the frequent finding of a low bone mass, decreased bone mineral content (BMC) and bone mineral density (BMD) [7]. In addition, the consumption of alcohol disrupts compact and trabecular bone microarchitecture. In general, alcohol negatively affects bone structure for several reasons. Bone remodeling is modified, mostly due to a decreased bone formation [8]. According to Broulik et al. [9], an inhibited bone formation, bone repair and decreased bone strength were identified in rats fed ethanol (7.6 g of 95% ethanol kg^−1^ b.w. daily for 3 months). Also, chronic alcohol abuse caused changes in calcium regulating hormones, mineral homeostasis, mechanical loading [10] and led to an accumulation of reactive oxygen species (ROS) [11]. Furthermore, NADPH oxidase expression was upregulated in osteoblasts of rats infused intragastrically with ethanol-containing liquid diets (12 g ethanol kg^−1^ daily for 4 weeks) [12]. These effects of alcohol may be caused by its high caloric density and bioactive properties [13]. Although alcohol belongs to the most commonly consumed neurotoxins by humans, there are no studies describing its influence on qualitative and quantitative 2D characteristics of the compact bone. Therefore, our study was aimed to determine in detail the microstructure of compact and trabecular bone tissues of mice after a subchronic peroral exposure to alcohol using 2D and 3D imaging methods.

## Methods

### Animals

In our experiment, ten clinically healthy 12-weeks-old Swiss mice (males) were used. The animals were obtained from the accredited experimental laboratory of the Pedagogical University in Cracow. The mice were randomly divided into two groups, of five animals each and were housed in individual flat-deck wire cages under a constant photoperiod of 12 h of daylight, temperature 20–24 °C and humidity 55% ± 10%. In the experimental group (group E) mice received a solution composed of 15% ethanol and water (1.7 g 100% ethanol kg^−1^ b.w. per day) for 8 weeks. The solution of alcohol in water has been made every day and it was administered orally to mice by a syringe (six doses of 50 µl 15% ethanol daily). Therefore, we have known the exact amount of daily fluid alcohol consumption. This corresponds to a consumption of six 0.5 dl of 40% ethanol or 2.5 l of 12° beer for 75 kg male adults. The second group without alcohol administration served as a control (group C).

### Procedures

At the end of treatment period, mice were put into a state of deep anesthesia by Vetbutal (Biowet, Poland) administration in the amount of 35 mg kg^−1^ b.w. and their femora were used for microscopical analyses. Each femur was macerated, degreased and embedded in epoxy resin Biodur (Günter von Hagens, Heidelberg, Germany) according to the methodology of Martiniakova et al. [14]. Transverse thin sections (70–80 μm) were prepared with a sawing microtome (Leitz 1600, Leica, Wetzlar, Germany) and affixed to glass slides with Eukitt (Merck, Darmstadt, Germany) [15]. The qualitative characteristics of the compact bone (2D analysis) were determined according to the internationally accepted classification systems of Enlow and Brown [16] and Ricqles et al. [17]. The quantitative parameters of the compact bone (2D analysis) were assessed using software Motic Images Plus 2.0 ML (Motic China Group Co., Ltd.) in all sides (*anterior, posterior, medialis, lateralis*) of thin sections. We measured area (μm^2^), perimeter (μm), maximum and minimum diameters (μm) of the primary osteons’ vascular canals, Haversian canals and secondary osteons in all sides of thin section in order to minimize statistical differences in the individual. Secondary osteons were distinguished from primary osteons (i.e., primary vascular canals) on the basis of the well-defined peripheral boundary (cement line) between secondary osteons and surrounding tissue. Cement line delimits secondary osteons and also Haversian canals and it is not found in primary osteons. In addition, secondary osteons intersect circumferential lamellae, whereas primary osteons do not [14, 17–19].

Quantitative 3D analyses of compact and trabecular bone tissues were determined using microcomputed tomography (μCT 50, Scanco Medical). Compact bone structure was analysed in a region of interest starting 5.2 mm from the end of the growth plate (distal epiphysis) and extending 1.5 mm at femoral midshaft. Following parameters were measured: relative bone volume (%), bone mineral density (BMD) (mg HA cm^−3^), relative bone volume without marrow cavity (%), bone surface without marrow cavity (mm^2^) and cortical bone thickness (mm). Trabecular bone structure was analysed in a region of interest starting 1.2 mm from the end of the growth plate (distal epiphysis) and extending 1.5 mm. We measured relative bone volume (%), trabecular number (mm^−1^), trabecular thickness (mm), trabecular separation (mm) and bone surface (mm^2^).

### Statistics

The measured values were expressed as mean ± standard deviation. The differences in quantitative characteristics of compact and trabecular bone tissues between mice from E and C groups were determined using an unpaired t-test (*p *< 0.05).

## Results

### Qualitative 2D analysis of compact bone tissue

**Fig. 1:**
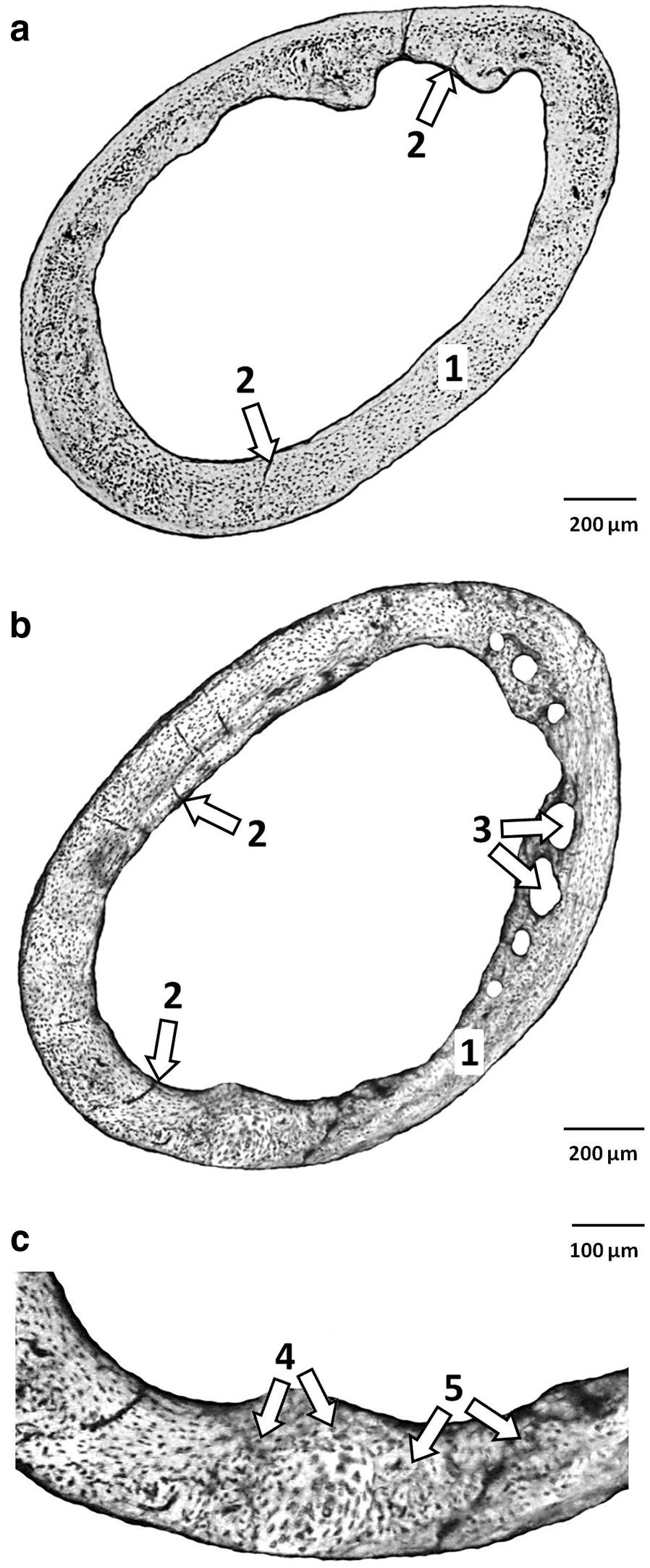
**a** Microstructure of compact bone tissue in mice from group C. 1—Non-vascular bone tissue. 2—primary vascular radial bone tissue. **b** Microstructure of compact bone tissue in mice from group E. 1—Non-vascular bone tissue, 2—primary vascular radial bone tissue. 3—Resorption lacunae. **c** Detailed view of *pars posterior* in mice from group E. 4—Vascular canals of primary osteons. 5—Secondary osteons delimited by the cement line with a central (Haversian) canal

The femora of mice from the C group had the following compact bone microstructure in common. The endosteal border consisted of a zone of non-vascular bone tissue (mainly in *anterior*, *medialis* and *lateralis* sides). This tissue included cellular lamellae and osteocytes. Also, primary vascular radial bone tissue (contained branching or non-branching vascular canals radiating from the marrow cavity) was observed in *pars posterior* of endosteal surface. In the middle part of the compact bone (in *anterior*, *lateralis* and *posterior* sides), irregular Haversian bone tissue was identified. This tissue consisted of Haversian systems (secondary osteons) which were scattered, isolated and relatively few in number. In addition, few resorption lacunae were found in *pars anterior*. Non-vascular bone tissue was observed in *pars medialis* of the central area of bone. Finally, the periosteal surface was formed by non-vascular bone tissue (Fig. 1a).

Mice from the E group displayed differences in *substantia compacta* compared to those from the C group (Fig. 1b). *Pars anterior* of the endosteal border was also formed by primary vascular radial bone tissue (not only by non-vascular bone tissue). Furthermore, more resorption lacunae (five times more) were identified in the middle part of *substantia compacta* (in *pars anterior* and *lateralis*).

### Quantitative 2D analysis of compact bone tissue

Altogether, 314 primary osteons’ vascular canals, 56 Haversian canals and 56 secondary osteons were measured in mice from both groups. The results are summarized in Table 1. We have found that all measured variables (area, perimeter, maximal and minimal diameters) of the primary osteons’ vascular canals had significantly higher values (*p *< 0.05) in mice from the E group. On the other hand, sizes of Haversian canals and secondary osteons did not differ significantly between mice from the E and C groups.

**Table 1:** Data of quantitative 2D analysis of compact bone tissue in mice from both groups

	Group	N	Area (μm^2^)	Perimeter (μm)	Max. diameter (μm)	Min. diameter (μm)
Primary osteons’ vascular canals	C	165	20.4533 ± 3.41	16.06 ± 1.355	2.6564 ± 0.296	2.402 ± 0.226
	E	149	30.1013 ± 6.14	19.496 ± 2.033	3.2987 ± 0.404	2.876 ± 0.362
	t-test		*p *< 0.05	*p *< 0.05	*p *< 0.05	*p *< 0.05
Haversian canals	C	26	22.06 ± 3.42	16.73 ± 1.32	2.81 ± 0.28	2.46 ± 0.27
	E	30	22.20 ± 4.86	16.65 ± 1.85	2.72 ± 0.36	2.54 ± 0.30
	t-test		NS	NS	NS	NS
Secondary osteons	C	26	243.63 ± 37.63	55.48 ± 4.47	9.38 ± 0.89	8.23 ± 0.64
	E	30	245.41 ± 48.99	55.71 ± 5.64	9.54 ± 1.09	8.14 ± 0.83
	t-test		NS	NS	NS	NS

*N* number of measured structures, *NS* non-significant differences

### Quantitative 3D analysis of compact bone tissue

**Fig. 2:**
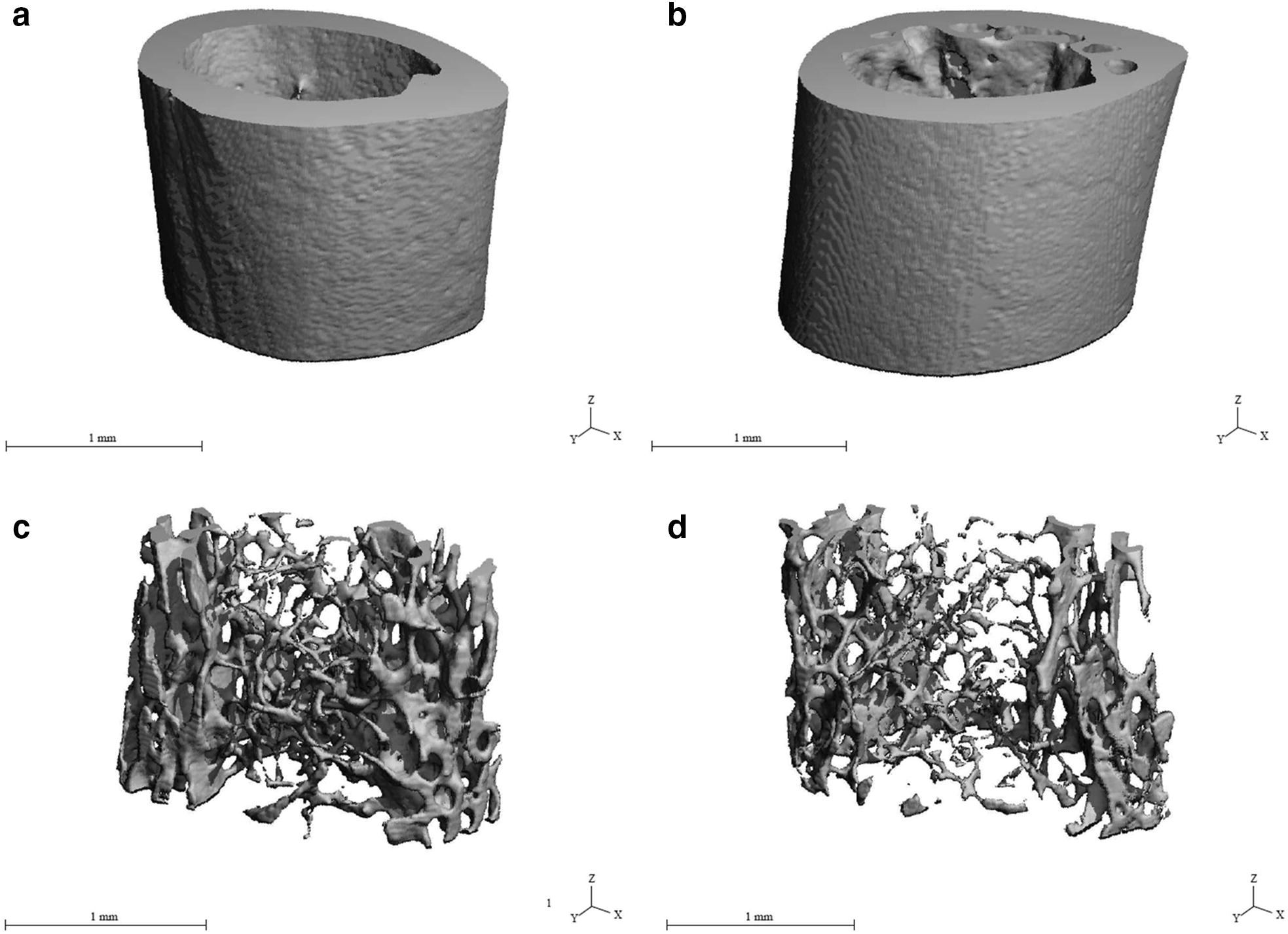
**a** Representative reconstructed 3D image of compact bone in mice from group C. **b** Representative reconstructed 3D image of compact bone in mice from group E. **c** Representative reconstructed 3D image of trabecular bone in mice from group C. **d** Representative reconstructed 3D image of trabecular bone in mice from group E

The values for relative bone volume and BMD were significantly decreased in mice from the group E. On the other hand, insignificant effects of subchronic alcohol consumption on relative bone volume without marrow cavity, bone surface and cortical bone thickness were observed. The results are summarized in Table 2. Representative reconstructed 3D images of the compact bone are illustrated in Fig. 2a, b.

**Table 2:** Data of quantitative 3D analysis of compact bone tissue in mice from both groups

Group	N	BV/TV (%)	BMD (mg HA cm^−3^)	BV/TV* (%)	Bs. (mm^2^)	Ct.Th. (mm)
C	5	0.509 ± 0.016	577.448 ± 27.439	0.958 ± 0.014	5.456 ± 1.356	0.195 ± 0.014
E	5	0.449 ± 0.022	513.406 ± 37.514	0.957 ± 0.003	4.704 ± 0.753	0.175 ± 0.010
t-test		*p *< 0.05	*p *< 0.05	NS	NS	NS

*n* number of measurements, *NS* non-significant differences, *BV/TV* relative bone volume, *BMD* bone mineral density, BV/TV* relative bone volume without marrow cavity, *Bs*. bone surface, *Ct.Th.* cortical bone thickness

### Quantitative 3D analysis of trabecular bone tissue

The values for relative bone volume, trabecular number, trabecular thickness and bone surface were significantly decreased (*p *< 0.05) in mice from the E group. On the contrary, the value for trabecular separation was significantly increased in these mice. The results are summarized in Table 3. Representative reconstructed 3D images of the trabecular bone are illustrated in Fig. 2c, d.

**Table 3:** Data of quantitative 3D analysis of trabecular bone tissue in mice from both groups

Group	N	BV/TV (%)	Tb.N. (mm^−1^)	Tb.Th. (mm)	Tb.Sp. (mm)	Bs. (mm^2^)
C	5	0.158 ± 0.010	4.616 ± 0.034	0.047 ± 0.001	0.210 ± 0.002	20.429 ± 3.516
E	5	0.078 ± 0.006	3.919 ± 0.134	0.035 ± 0.001	0.253 ± 0.011	13.417 ± 0.872
t-test		*p *< 0.05	*p *< 0.05	*p *< 0.05	*p *< 0.05	*p *< 0.05

*n* number of measurements, *BV/TV* relative bone volume, *Tb*.*N*. trabecular number, *Tb.Th.* trabecular thickness, *Tb.Sp.* trabecular separation, *Bs*. bone surface

## Discussion

The results of qualitative 2D analysis of the compact bone in mice from the C group are consistent with those of other researchers [16, 20, 21]. However, subchronic exposure to ethanol leads to several changes such as the presence of primary vascular radial bone tissue in *pars anterior* of endosteal border and a higher number of resorption lacunae (increased porosity) in the middle part of *substantia compacta.* These differences can be caused by inhibition of periosteal and endosteal bone formation due to alcohol administration [22]. The results by Rocco et al. [23] showed that alcohol metabolized by CYP2E1 is responsible for a formation of reactive oxygen species (ROS), such as hydrogen peroxide and superoxide ions which enhance bone resorption [24] and osteoclastogenesis [25]. During growth, the entire bone is moved and repositioned in a progressive anterior direction [26]. Also, this bone region is characteristic by a higher degree of mineralization [27]. According to Sivaraj and Adams [28], vascularization constitutes the first phase of ossification: the blood vessels invade the cartilage and later produce resorption via the osteoclasts originating from the nearby vessels. In the same way, vascular neoformation is the first event in the repair of fractures or bone regeneration.

Our results also revealed significantly increased sizes of primary osteons’ vascular canals in mice from the E group. Primary osteons’ vascular canals contain blood vessels which provide nutrition for the bone [29]. Pries et al. [30] showed that blood vessels can adapt its structure (vascular remodeling) in response to continuous functional changes. Vasodilation of these vascular canals in mice exposed to alcohol could be associated with deleterious effect of alcohol on blood vessels. According to Palaparthy [31], alcohol has a significant effect on cardiovascular system including peripheral vasodilation. Generally, angiogenesis is regulated by various factors, including signaling through vascular endothelial growth factor (VEGF) receptors [32]. In general, hypoxia can be originated by the presence of alcohol in the liver [33]. Hypoxic conditions can also potently stimulate a formation of an extensive vascular network [34]. On the other hand, non-significant changes in the size of Haversian canals (which also contain blood vessels) were observed in mice from E group. It is generally known that the structure of primary and secondary osteons is different. Haversian canals are surrounded by a cement line which is not found in primary osteons [14, 17–19]. We suppose that the cement line is a main reason for different results in histomorphometry of both canals.

Quantitative 3D analysis of the compact bone discovered significantly lower values for relative bone volume and BMD in mice administered alcohol. Decreased BMD and relative bone volume were also documented in other studies with alcohol-fed mice (36% ethanol Lieber-DeCarli diet for 78 days) [35] and rats (35% ethanol Lieber-DeCarli liquid diet for 42 days) [36]. According to Wezeman et al. [37], chronic alcohol exposure can stimulate metabolism of adipocytes resulting in triglyceride accumulation in diaphyseal marrow of the bone. Also, other cellular changes in the bone marrow and endocortical surface of the bone caused by alcohol consumption lead to the disruption of bone remodeling involving reduction of the number and activity of basic multicellular units [6]. Therefore, decreased bone formation rate followed by a low bone mass and a decreased BMD are generally identified in alcoholics.

We found significantly decreased values for relative bone volume, trabecular number, trabecular thickness and bone surface in mice from the E group. These results confirmed the literature [38–40] showing that alcohol intake reduces the trabecular bone volume and leads to trabecular thinning. According to Turner et al. [41], alcohol also impairs the effects of growth hormone (an important physiological regulator of bone growth) on tibial growth and trabecular bone formation. Furthermore, alcohol decreases concentration of leptin [39]. Leptin has a direct anabolic effect on the bone that leads to the decline of osteoblastic differentiation [42]. Therefore, evident changes in morphometry of the trabecular bone could be associated with these aspects.

In summary, alcohol decreased not only bone volume and density of the compact bone, but it also reduced trabecular bone volume and leads to trabecular thinning in our male mice. In men, chronic heavy alcohol consumption also decreased BMD in different skeletal sites, including femur [7, 43, 44]. Also, decreased cortical thickness, trabecular thickness and volume were identified in black male patients suffering from alcohol-induced bone disease and pancreatitis [45]. Vasodilation of primary osteons’ vascular canals and increased porosity in the compact bone were observed in male mice. In general, bone microstructure of adult humans is composed mainly of dense Haversian bone tissue with a quite dense concentration of secondary osteons. Primary osteons are only occasionally found in the bones [19, 46]. Therefore, the information about vasodilation of primary osteons’ vascular canals in human alcoholics is still absent. Increased porosity (including increased bone resorption parameters) was also identified in osteoporotic males [47]. Heavy alcohol consumption is known to induce secondary osteoporosis in both females and males. It follows that almost all changes described in our study are observed in humans consuming alcohol.

## Conclusions

Subchronic peroral exposure to alcohol at the dose used in our study affected both compact and trabecular bone tissues microstructure. In the compact bone, increased porosity, significantly higher values for primary osteons’ vascular canals and decreased values for relative bone volume and bone mineral density were identified. In the trabecular bone, decreased values for bone volume, trabecular number, trabecular thickness and bone surface were documented. Our study supports previously published results showing that excess alcohol consumption is damaging for both compact and trabecular bone tissues. However, it provides the first information related to changes in qualitative and quantitative 2D characteristics of the compact bone of mice subchronically exposed to ethanol.

## Abbreviations

BMC: bone mineral content
BMD: bone mineral density
b.w.: body weight
g: gram
kg: kilogram
ROS: reactive oxygen species
NADPH: nicotinamide adenine dinucleotide phosphate
µm: micrometre
μCT: microcomputed tomography
BV/TV: relative bone volume
BV/TV*: relative bone volume without marrow cavity
Bs.: bone surface without marrow cavity
Ct.Th.: cortical bone thickness
HA: hydroxyapatite
Tb.N.: trabecular number
Tb.Sp.: trabecular separation
Tb.Th.: trabecular thickness
mm: millimetre
N: number of measurements
CYP2E1: cytochrome P450 2E1
VEGF: vascular endothelial growth factor
NS: non-significant differences
SD: standard deviation
